# Avid ^18^F-FDG Uptake in Idiopathic Tumoral Calcinosis Mimicking Lymph Node Metastasis

**DOI:** 10.3390/diagnostics7040060

**Published:** 2017-12-13

**Authors:** Jesper Strandberg, Helle D. Zacho

**Affiliations:** 1 Department of Nuclear Medicine, Aalborg University Hospital, Hobrovej 18-22, DK-9000 Aalborg, Denmark; h.zacho@rn.dk; 2 Department of Clinical Medicine, Aalborg University, DK-9000 Aalborg, Denmark

**Keywords:** ^18^F-Fludeoxyglucose positron emission tomography/computed tomography (^18^F-FDG PET/CT), chronic renal failure, tumoral calcinosis

## Abstract

Tumoral calcinosis is a benign condition characterized by periarticular calcified lesions that is frequently observed in patients with chronic renal failure. Tumoral calcinosis often presents with subcutaneous masses and joint swelling. We present a case of tumoral calcinosis with dramatically increased ^18^F-fluoro-2-deoxy-d-glucose (^18^F-FDG) uptake on positron emission tomography/computed tomography (PET/CT) that mimicked lymphoma or lymph node metastases.

## 1. Introduction

Tumoral calcinosis may occur in all tissues [1]. This condition is a rare complication of chronic renal failure, and the genesis of the disease is related to high levels of calcium and phosphorus products and/or advanced secondary hyperparathyroidism after long-term dialysis [2,3]. High PTH (parathyroid hormone) can cause very high levels of plasma calcium. Additionally, extracellular phosphate concentration is often increased, secondary to kidney malfunction; hence, phosphate cannot be properly excreted. Therefore, the body fluids become supersaturated with calcium and phosphate, and calcium phosphate crystals begin to be deposited in tissue [4].

Patients often present with localized swelling around joints and reduced mobility. The most common locations of tumoral calcinosis are the hips, elbows, shoulders, feet, and wrists [5]. The treatment of tumoral calcinosis involves eliminating the underlying causes of either hyperphosphatemia or hypercalcemia and may include renal transplantation or parathyroidectomy [6].

^18^F-Fludeoxyglucose positron emission tomography/computed tomography (^18^F-FDG PET/CT) is used for the evaluation and initial staging of lymphomas. This method can detect more lesions than CT-scans can due to its ability to detect metabolic changes before structural changes become visible [7].

## 2. Case Report

The study subject is a 57-year-old man with chronic terminal kidney disease due to focal segmental glomerulosclerosis (FSGS) diagnosed in 1997, and secondary/tertiary hyperparathyroidism with severe hyperphosphatemia. His blood values remained stable for 18 years after the diagnosis of FSGS. However, since 2015 he developed increasing uremia with serum creatinine rose from 350 to 550 corresponding to a glomerular filtration rate of approximately 10 mL/min, but dialysis was not yet required. During the same time span PTH increased from 25 to 100 pmol/L with concomitant fluctuating values for phosphate (2.5–3.5 mmol/L). The total plasma Ca^2+^ level remained within the normal range during the entire period from diagnosis to present.

The patient presented with complaints of rapid development of palpable subcutaneous swellings in his right shoulder, axilla, and on the right side of his neck with neither pain nor sensory disturbances for 7–10 days. Apart from fatigue, no B-symptoms, fever or signs of upper respiratory tract infection were present, but several palpable lymph nodes on the right side of the neck along the sternocleidomastoid muscle and in the right axilla were noted. Blood samples revealed slightly elevated infection parameters. The patient was referred to the Department of Hematology at Aalborg University Hospital, where malignant lymphoma was suspected.

**Figure 1:**
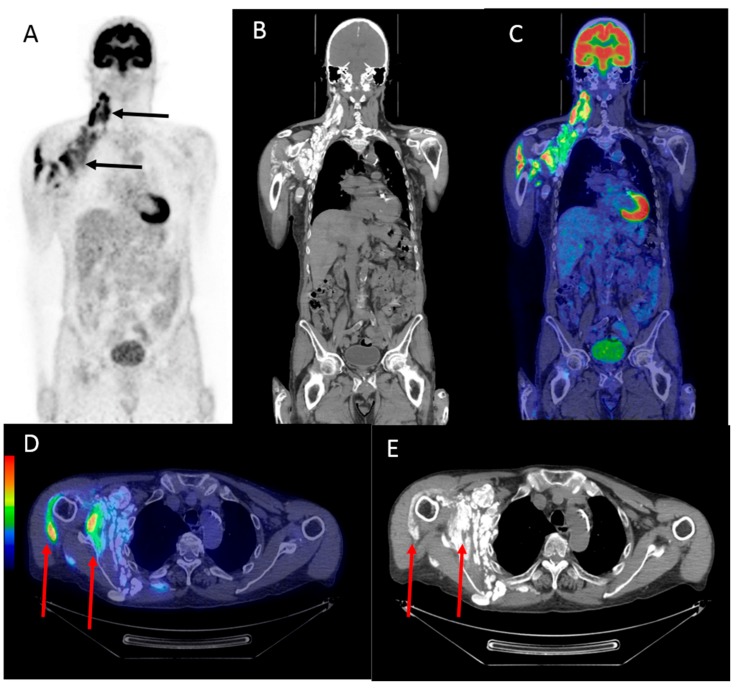
The maximum intensity projection (MIP) of the ^18^F-Fludeoxyglucose (^18^F-FDG) positron emission tomography (PET) image in the anterior view (**A**) showing multiple lesions with high ^18^F-FDG uptake located on the right side of the neck, around the right shoulder, and in the right axillar region (black arrows). A coronal computed tomography (CT) image (**B**) and a fused coronal image (**C**) of the head, neck, and upper body showing the same regions with high ^18^F-FDG uptake seen in the anterior view (**A**). Fused transversal images (**D**) and transversal CT images (**E**) showing the intramuscular high ^18^F-FDG uptake in the right axillary region (red arrows).

A lymph node biopsy from the right side of the neck revealed cystic material with calcification and no malignant cells. The patient then underwent a ^18^F-FDG PET/CT scan, which showed multiple sites of severe calcification of the right side of the neck and in the right axilla with increased ^18^F-FDG uptake that mimicked multiple lymph node metastases (Figure 1). Additional avid ^18^F-FDG uptake was observed in the shoulder muscles. To reach a final diagnosis, a lymphadenectomy was performed. Histopathology revealed fat and connective tissue as well as calcifications with surrounding foreign body reaction/inflammation. There were no signs of malignancy or premalignacy but histology revealed multiple calcifications, most likely due to the disturbed calcium-phosphate balance resulting from the patient’s chronic renal failure. Additional culturing of the cyst content did not reveal any infectious agents. Apart from physiotherapy to improve the minor movement limitations of his right arm, no other treatment was required.

## 3. Discussion

^18^F-FDG uptake in tumoral calcinosis has only been sporadically described. Okuyama et al. reported a case of tumoral calcinosis in the right cheek with increased ^18^F-FDG uptake [8]. Additionally, Liu reported a case of tumoral calcinosis in the neck, elbow, wrist, hand, thighs, perineum, and paraspinal muscles with increased uptake on ^18^F-FDG PET/CT [5]. The raised PTH causing the disturbances in calcium-homeostasis and subsequent tumoral calcinosis can also be secondary to a PTH-producing tumor [9].

In the present case the patient suffered from chronic renal failure, and the histology of the removed lymph nodes revealed extensive foreign body reaction/inflammation. This may explain the increased ^18^F-FDG-uptake in and around the calcified areas. During chronic stages of soft tissue calcification less inflammation is presented and subsequently less—if any—^18^F-FDG-uptake would be expected during a chronic situation. Tumoral calcinosis is a rare condition and may mimic lymphoma or lymph node metastases when it presents as palpable masses; this is further supported when the masses exhibit high ^18^F-FDG uptake. However, the association of calcifications in the muscles in a patient with chronic renal failure should raise the attention of the physician interpreting the PET/CT images.
